# Supplementary material for: Empirical methods for controlling false positives and estimating confidence in ChIP-Seq peaks
Source: BMC Bioinformatics. 2008 Dec 5;9:523. doi: 10.1186/1471-2105-9-523 (PMC2628906; doi:10.1186/1471-2105-9-523)
Supplement: Additional File 1 — A variety of html documents from the USeq web site detailing the available applications, their best usage, output file type descriptions, command line menus, etc. [file 1471-2105-9-523-S1.zip › USeqUserGuides/JavaDoc.zip!/JavaDoc/gata/plotter/AlignPanel.html]

AlignPanel


|  |  |  |
| --- | --- | --- |
| **Overview** \| **Package** \| **Class** \| **Use** \| **Tree** \| **Deprecated** \| **Index** \| **Help** | |  |
| PREV CLASS   **NEXT CLASS** | **FRAMES**    **NO FRAMES**     **All Classes** |  |
| SUMMARY: NESTED \| FIELD \| CONSTR \| METHOD | DETAIL: FIELD \| CONSTR \| METHOD |  |


---


## gata.plotter Class AlignPanel

```
java.lang.Object
  java.awt.Component
      java.awt.Container
          javax.swing.JComponent
              javax.swing.JPanel
                  gata.plotter.AlignPanel
```

**All Implemented Interfaces:**: java.awt.image.ImageObserver, java.awt.MenuContainer, java.io.Serializable, javax.accessibility.Accessible

---

``` public class AlignPanel extends javax.swing.JPanel ```

**Author:**
:   Nix
    Panel holding the DNA box-line-box alignment

**See Also:**: Serialized Form

---

**Nested Class Summary**

**Nested classes/interfaces inherited from class javax.swing.JComponent**

`javax.swing.JComponent.AccessibleJComponent`


**Field Summary**

**Fields inherited from class javax.swing.JComponent**

`TOOL_TIP_TEXT_KEY, UNDEFINED_CONDITION, WHEN_ANCESTOR_OF_FOCUSED_COMPONENT, WHEN_FOCUSED, WHEN_IN_FOCUSED_WINDOW`

**Fields inherited from class java.awt.Component**

`BOTTOM_ALIGNMENT, CENTER_ALIGNMENT, LEFT_ALIGNMENT, RIGHT_ALIGNMENT, TOP_ALIGNMENT`

**Fields inherited from interface java.awt.image.ImageObserver**

`ABORT, ALLBITS, ERROR, FRAMEBITS, HEIGHT, PROPERTIES, SOMEBITS, WIDTH`


**Constructor Summary**

`AlignPanel(GATAParams params)`


| **Method Summary** | |
| --- | --- |
| `static void` | `createShapes(Alignment[] alignments, double refX, double refY, double compX, double compY, double ntLenBig, double C, double D)` |
| `int` | `getAlignFrameHeight()` |
| `int` | `getAlignFrameWidth()` |
| `Alignment[]` | `getAlignments()` |
| `int` | `getHeightBuffImage()` |
| `javax.swing.JViewport` | `getViewPort()` |
| `Alignment[]` | `getVisableAlignments()` |
| `int` | `getWidthBuffImage()` |
| `void` | `makeAllShapes(int width, int height)` |
| `java.awt.image.BufferedImage` | `makeBufferedImage(double scale, boolean saveVisible)`             Generates a BufferedImage from the panel. |
| `void` | `makeVisableAlignments()`             Resets an array of visable alignments |
| `void` | `paintComponent(java.awt.Graphics g)` |
| `void` | `redrawGlyphPanel(AnnoSpecParams asp)` |
| `void` | `setAlignFrameWidth(int i)` |
| `void` | `setConsole(Console console)` |
| `void` | `setDrawCompBox(boolean drawCompBox)` |
| `void` | `setMaxScore(double max)` |
| `void` | `setMinScore(double min)` |
| `void` | `setPresenceOfGFFs()` |
| `void` | `setToolsRef(ToolsFrame aTF)` |
| `void` | `setViewPort(javax.swing.JViewport viewport)` |
| `void` | `zoom(int zScalar)` |

**Methods inherited from class javax.swing.JPanel**

`getAccessibleContext, getUI, getUIClassID, setUI, updateUI`

**Methods inherited from class javax.swing.JComponent**

`addAncestorListener, addNotify, addVetoableChangeListener, computeVisibleRect, contains, createToolTip, disable, enable, firePropertyChange, firePropertyChange, firePropertyChange, getActionForKeyStroke, getActionMap, getAlignmentX, getAlignmentY, getAncestorListeners, getAutoscrolls, getBorder, getBounds, getClientProperty, getComponentPopupMenu, getConditionForKeyStroke, getDebugGraphicsOptions, getDefaultLocale, getFontMetrics, getGraphics, getHeight, getInheritsPopupMenu, getInputMap, getInputMap, getInputVerifier, getInsets, getInsets, getListeners, getLocation, getMaximumSize, getMinimumSize, getNextFocusableComponent, getPopupLocation, getPreferredSize, getRegisteredKeyStrokes, getRootPane, getSize, getToolTipLocation, getToolTipText, getToolTipText, getTopLevelAncestor, getTransferHandler, getVerifyInputWhenFocusTarget, getVetoableChangeListeners, getVisibleRect, getWidth, getX, getY, grabFocus, isDoubleBuffered, isLightweightComponent, isManagingFocus, isOpaque, isOptimizedDrawingEnabled, isPaintingTile, isRequestFocusEnabled, isValidateRoot, paint, paintImmediately, paintImmediately, print, printAll, putClientProperty, registerKeyboardAction, registerKeyboardAction, removeAncestorListener, removeNotify, removeVetoableChangeListener, repaint, repaint, requestDefaultFocus, requestFocus, requestFocus, requestFocusInWindow, resetKeyboardActions, reshape, revalidate, scrollRectToVisible, setActionMap, setAlignmentX, setAlignmentY, setAutoscrolls, setBackground, setBorder, setComponentPopupMenu, setDebugGraphicsOptions, setDefaultLocale, setDoubleBuffered, setEnabled, setFocusTraversalKeys, setFont, setForeground, setInheritsPopupMenu, setInputMap, setInputVerifier, setMaximumSize, setMinimumSize, setNextFocusableComponent, setOpaque, setPreferredSize, setRequestFocusEnabled, setToolTipText, setTransferHandler, setVerifyInputWhenFocusTarget, setVisible, unregisterKeyboardAction, update`

**Methods inherited from class java.awt.Container**

`add, add, add, add, add, addContainerListener, addPropertyChangeListener, addPropertyChangeListener, applyComponentOrientation, areFocusTraversalKeysSet, countComponents, deliverEvent, doLayout, findComponentAt, findComponentAt, getComponent, getComponentAt, getComponentAt, getComponentCount, getComponents, getComponentZOrder, getContainerListeners, getFocusTraversalKeys, getFocusTraversalPolicy, getLayout, getMousePosition, insets, invalidate, isAncestorOf, isFocusCycleRoot, isFocusCycleRoot, isFocusTraversalPolicyProvider, isFocusTraversalPolicySet, layout, list, list, locate, minimumSize, paintComponents, preferredSize, printComponents, remove, remove, removeAll, removeContainerListener, setComponentZOrder, setFocusCycleRoot, setFocusTraversalPolicy, setFocusTraversalPolicyProvider, setLayout, transferFocusBackward, transferFocusDownCycle, validate`

**Methods inherited from class java.awt.Component**

`action, add, addComponentListener, addFocusListener, addHierarchyBoundsListener, addHierarchyListener, addInputMethodListener, addKeyListener, addMouseListener, addMouseMotionListener, addMouseWheelListener, bounds, checkImage, checkImage, contains, createImage, createImage, createVolatileImage, createVolatileImage, dispatchEvent, enable, enableInputMethods, firePropertyChange, firePropertyChange, firePropertyChange, firePropertyChange, firePropertyChange, getBackground, getBounds, getColorModel, getComponentListeners, getComponentOrientation, getCursor, getDropTarget, getFocusCycleRootAncestor, getFocusListeners, getFocusTraversalKeysEnabled, getFont, getForeground, getGraphicsConfiguration, getHierarchyBoundsListeners, getHierarchyListeners, getIgnoreRepaint, getInputContext, getInputMethodListeners, getInputMethodRequests, getKeyListeners, getLocale, getLocation, getLocationOnScreen, getMouseListeners, getMouseMotionListeners, getMousePosition, getMouseWheelListeners, getName, getParent, getPeer, getPropertyChangeListeners, getPropertyChangeListeners, getSize, getToolkit, getTreeLock, gotFocus, handleEvent, hasFocus, hide, imageUpdate, inside, isBackgroundSet, isCursorSet, isDisplayable, isEnabled, isFocusable, isFocusOwner, isFocusTraversable, isFontSet, isForegroundSet, isLightweight, isMaximumSizeSet, isMinimumSizeSet, isPreferredSizeSet, isShowing, isValid, isVisible, keyDown, keyUp, list, list, list, location, lostFocus, mouseDown, mouseDrag, mouseEnter, mouseExit, mouseMove, mouseUp, move, nextFocus, paintAll, postEvent, prepareImage, prepareImage, remove, removeComponentListener, removeFocusListener, removeHierarchyBoundsListener, removeHierarchyListener, removeInputMethodListener, removeKeyListener, removeMouseListener, removeMouseMotionListener, removeMouseWheelListener, removePropertyChangeListener, removePropertyChangeListener, repaint, repaint, repaint, resize, resize, setBounds, setBounds, setComponentOrientation, setCursor, setDropTarget, setFocusable, setFocusTraversalKeysEnabled, setIgnoreRepaint, setLocale, setLocation, setLocation, setName, setSize, setSize, show, show, size, toString, transferFocus, transferFocusUpCycle`

**Methods inherited from class java.lang.Object**

`equals, getClass, hashCode, notify, notifyAll, wait, wait, wait`

**Constructor Detail**

### AlignPanel

```
public AlignPanel(GATAParams params)
```


**Method Detail**

### setToolsRef

```
public void setToolsRef(ToolsFrame aTF)
```

---


### setPresenceOfGFFs

```
public void setPresenceOfGFFs()
```

---


### zoom

```
public void zoom(int zScalar)
```

---


### makeBufferedImage

```
public java.awt.image.BufferedImage makeBufferedImage(double scale,
                                                      boolean saveVisible)
```

:   Generates a BufferedImage from the panel. Can be scaled or cropped to visible.

---


### redrawGlyphPanel

```
public void redrawGlyphPanel(AnnoSpecParams asp)
```

---


### makeAllShapes

```
public void makeAllShapes(int width,
                          int height)
```

---


### createShapes

```
public static void createShapes(Alignment[] alignments,
                                double refX,
                                double refY,
                                double compX,
                                double compY,
                                double ntLenBig,
                                double C,
                                double D)
```

---


### paintComponent

```
public void paintComponent(java.awt.Graphics g)
```

:   **Overrides:**: `paintComponent` in class `javax.swing.JComponent`

---


### makeVisableAlignments

```
public void makeVisableAlignments()
```

:   Resets an array of visable alignments

---


### setMinScore

```
public void setMinScore(double min)
```

---


### setMaxScore

```
public void setMaxScore(double max)
```

---


### setConsole

```
public void setConsole(Console console)
```

---


### getVisableAlignments

```
public Alignment[] getVisableAlignments()
```

---


### setViewPort

```
public void setViewPort(javax.swing.JViewport viewport)
```

---


### setAlignFrameWidth

```
public void setAlignFrameWidth(int i)
```

---


### getAlignFrameHeight

```
public int getAlignFrameHeight()
```

---


### getAlignFrameWidth

```
public int getAlignFrameWidth()
```

---


### getHeightBuffImage

```
public int getHeightBuffImage()
```

---


### getWidthBuffImage

```
public int getWidthBuffImage()
```

---


### getViewPort

```
public javax.swing.JViewport getViewPort()
```

---


### setDrawCompBox

```
public void setDrawCompBox(boolean drawCompBox)
```

---


### getAlignments

```
public Alignment[] getAlignments()
```


---


|  |  |  |
| --- | --- | --- |
| **Overview** \| **Package** \| **Class** \| **Use** \| **Tree** \| **Deprecated** \| **Index** \| **Help** | |  |
| PREV CLASS   **NEXT CLASS** | **FRAMES**    **NO FRAMES**     **All Classes** |  |
| SUMMARY: NESTED \| FIELD \| CONSTR \| METHOD | DETAIL: FIELD \| CONSTR \| METHOD |  |


---
